# Advances in non‐invasive imaging for dermatofibrosarcoma protuberans: A review

**DOI:** 10.1111/ajd.14366

**Published:** 2024-10-03

**Authors:** Yanci A. Algarin, Anika Pulumati, Jiali Tan, Nathalie C. Zeitouni

**Affiliations:** ^1^ Eastern Virginia Medical School Norfolk Virginia USA; ^2^ University of Missouri‐Kansas City School of Medicine Kansas City Missouri USA; ^3^ Albany Medical College Albany New York USA; ^4^ Medical Dermatology Specialists University of Arizona COM Phoenix Phoenix Arizona USA

**Keywords:** computed tomography, dermatofibrosarcoma protuberans, magnetic resonance imaging, non‐invasive imaging, ultrasound

## Abstract

Dermatofibrosarcoma protuberans (DFSP) is a rare soft tissue sarcoma characterized by an asymmetric, infiltrative growth pattern and a high risk of local recurrence. This study aims to evaluate the effectiveness of various imaging modalities in the assessment and management of DFSP. Nine imaging modalities were reviewed including: Ultrasound (US), High‐Frequency Doppler Ultrasound (HFUS), Computed tomography (CT), Positron emission tomography–computed tomography (PET‐CT), and Magnetic Resonance Imaging (MRI), High‐resolution‐MRI (HR‐MRI), Magnetic Resonance Spectroscopy (MRS), Optical Coherence Tomography (OCT), and Dermatoscopy. Imaging is mainly used for preoperative assessment and surgical planning, not routine diagnosis. US is effective for initial evaluations, demonstrating superior ability in detecting muscle invasion and defining tumour boundaries (sensitivity – 81.8%, specificity – 100%). MRI is valuable for preoperative evaluation, surgical planning, and monitoring DFSP recurrence. It more accurately assesses tumour depth than palpation, with a sensitivity of 67% and specificity of 100%, but was inferior when compared to US. CT is utilized in cases of suspected bone involvement or pulmonary metastasis. For advanced or recurrent DFSP, PET‐CT helps manage treatment responses and imatinib therapy. Emerging technologies like MRS and OCT show potential in improving diagnostic accuracy and defining surgical margins, though more data are needed. US, MRI, and CT are the primary imaging modalities for DFSP. Emerging technologies like HR‐MRI, PET‐CT, MRS, and OCT hold promise for refining diagnostic and management strategies. Integrating multiple technologies could enhance management, particularly in atypical or aggressive cases. Further studies are required to refine imaging protocols and improve DFSP outcomes.

## INTRODUCTION

Dermatofibrosarcoma protuberans (DFSP) is a rare soft tissue sarcoma of the dermis, characterized by its locally aggressive and infiltrative nature. It can invade fascial planes, affecting bones and muscles. While metastasis is infrequent, it has the propensity to recur locally.^1^

In recent years, there has been a surge in the use of non‐invasive imaging methods among dermatologists and other providers to aid in the assessment and management of DFSP. These techniques include ultrasound (US), high‐frequency ultrasound (HFUS), computed tomography (CT), positron emission tomography‐computed tomography (PET‐CT), magnetic resonance imaging (MRI), high‐resolution MRI (HR‐MRI), magnetic resonance spectroscopy (MRS), optical coherence tomography (OCT), dermatoscopy. This review aims to evaluate the effectiveness of these imaging tools in managing DFSP, highlighting their advantages, limitations, and overall effectiveness.

## DERMATOFIBROSARCOMA PROTUBERANS: AN OVERVIEW

Dermatofibrosarcoma protuberans accounts for about 0.1% of all cancers and most commonly occurs in young adults.^1^, ^2^ There are several subtypes, including Bednar, myxoid, and fibrosarcomatous.^3^ Clinically, DFSP initially presents as a slow‐growing, flesh‐coloured, asymptomatic nodule. As it progresses, it may evolve into a painful and even ulcerative lesion.^4^ Due to its initial presentation, DFSP can be mistaken for other benign skin lesions, such as dermatofibromas or epidermoid cysts, which might delay diagnosis. Definitive diagnosis is established through histopathological examination of a biopsy specimen with immunohistochemical staining and possible molecular diagnostic testing. Histologically, DFSP is characterized by spindle cells in a storiform pattern.^5^ Complete surgical resection with wide local excision (WLE) or Mohs micrographic surgery (MMS) is still the primary treatment for DFSP. WLE often requires wide margins (>3–5 cm) resulting in large postoperative defects. Conversely, MMS may be time‐consuming. Non‐invasive imaging may help overcome some of these constraints by allowing a preoperative visualization of the tumour's margins and infiltration depth. This can potentially help clinicians in minimizing the defect size, improve healing, and enhance surgical outcomes.^6^

## METHODS

### Search strategy and data sources

We performed a comprehensive search via PubMed and Google Scholar from 1990 to 2024 using the following search terms and keywords: “Dermatofibrosarcoma protuberans” AND one of the following search terms: “Ultrasound” OR “High frequency doppler ultrasound” OR “Computed Tomography” OR “Positron emission tomography–computed tomography” OR “Magnetic resonance imaging” OR “Magnetic resonance spectroscopy” OR “Optical coherence tomography” OR “Dermatoscopy”. Our search was limited to studies on humans and English‐language publications. Other references were identified from the references of relevant articles.

### Inclusion criteria

Our study included peer‐reviewed journal articles on non‐invasive imaging for diagnosing and managing DFSP. Inclusion criteria required detailed information on imaging modalities, while exclusion criteria encompassed unrelated articles, those with insufficient information, and duplicates.

## NON‐INVASIVE IMAGING MODALITIES

### US

US, including HFUS, serves as a quick, accessible, and cost‐effective initial imaging tool for superficial soft‐tissue masses.^6^ For DFSP, US is performed in cases with atypical clinical appearance or to determine tumour extent and depth.^7^ DFSP typically presents in the US as oval‐shaped, hypoechoic masses featuring pseudopodia‐like deep protrusions, well‐defined lobulated borders, and occasional variations such as masses with mixed hypoechoic and hyperechoic regions.^7^, ^8^, ^9^, ^10^, ^11^, ^12^, ^13^, ^14^, ^15^ These hypoechoic areas often reflect high cellularity with spindle cells in storiform patterns, whereas hyperechoic areas are associated with a mix of DFSP cells and fibrous tissue infiltrating subcutaneous fat.^8^, ^16^ Mild to moderate acoustic enhancement is commonly observed.^8^, ^16^

A recent large case series (*n* = 30) confirmed the reliability of US patterns in DFSP, highlighting four characteristic patterns of invasion: (1) oval hypoechoic with subtle, superficial subcutaneous invasion (*n* = 3, 10%), (2) oval hypoechoic with finger‐like projections (*n* = 6, 20%); (3) oval hypoechoic with finger‐like projections and posterior hyperechoic area in subcutaneous tissue or deeper (*n* = 16, 53.3%); (4) mixed invasive pattern with both hypoechoic and hyperechoic areas (*n* = 5, 16.7%).^15^ Similar to previous studies, there was a good correlation between US images and histopathological features.^8^, ^13^, ^14^, ^15^, ^16^ US and histopathological measurements of tumour depth demonstrated strong concordance (7.3 ± 4.0 mm by ultrasound vs. 8.3 ± 4.2 mm by histopathology). Furthermore, the US demonstrated a high sensitivity (81.8%) and a very high specificity (100%) in detecting muscle or muscle fascia invasion, with positive and negative predictive values of 83.3%.^15^

HFUS offers advanced capabilities for blood flow estimation and resolution, revealing variable vascularization in DFSP that ranges from absent to moderate, typically around the lesion's periphery.^9^, ^16^, ^17^ This technique proves particularly useful in excluding non‐vascular lesions like cysts and lipomas, and differentiating DFSP from vascular benign tumours such as hemangiomas and arteriovenous malformations (AVMs).^8^, ^18^ A retrospective case series comparing HFUS with MRI and histological findings highlighted its superiority in accurately determining tumour margins, often showing larger tumour diameters than MRI, which occasionally fails to detect deep infiltration or the tumour altogether.^19^ Moreover, HFUS can be administered during initial dermatological consultations, offering a significant time advantage over MRI, which averaged a 25‐day delay.^19^ HFUS's safety and ease of use also extend to paediatric cases, as demonstrated in a four‐year‐old patient initially suspected of having a hemangioma or AVM, where HFUS was instrumental in diagnosing DFSP.^18^

Overall, US's accessibility, cost‐effectiveness, and ability to provide accurate information on tumour extension makes it a valuable pre‐operative tool for DFSP. Clinically, US can be used to aid the initial assessment of DFSP, especially for lesions with atypical presentations. If US features suggest DFSP, a biopsy should be performed to confirm the diagnosis.^6^ US can also be used for preoperative evaluation and surgical planning.^7^, ^9^, ^10^, ^11^, ^12^, ^13^, ^14^, ^15^ However, the nonspecific US features of DFSP (oval, well‐defined lesions, sometimes with lobulated margins) impose some limitations.^20^ Clinicians should have a thorough understanding of the diverse US imaging features of DFSP. When assessing a soft tissue mass for a differential diagnosis, it is important to consider all available information, including demographic details, laboratory results, and findings from various imaging methods. More studies are needed to determine the usefulness of US for assessing lateral tumour extension, and to compare its efficacy with MRI for DFSP.

### CT

CT is generally reserved for recurrent or aggressive cases of DFSP in which underlying bone involvement is suspected.^6^, ^21^, ^22^ CT is used for identifying the anatomical location, determining size and shape of the lesion, assessing underlying bone involvement or calcifications, and staging for distant metastasis.^6^ DFSP typically appears on CT as a well‐demarcated nodular structure with a density resembling that of skeletal muscle.^21^, ^23^, ^24^ In a study with nine DFSP patients, CT scans conducted before and after contrast administration revealed iso‐ or hypo‐density relative to muscle in seven lesions, with inhomogeneous moderate to progressive enhancement noted during the arterial phase in contrast‐enhanced CT.^25^ Further analysis by Zhang et al.^21^ on histopathologically confirmed DFSP cases showed that unenhanced CT images exhibited 19 homogenous isodense lesions, whereas contrast‐enhanced CT revealed diverse enhancement patterns, including intermediate and marked nonhomogeneous (13 lesions), intermediate homogeneous (4 lesions), and mild heterogeneous (2 lesions). No calcifications were detected in these lesions, aligning with the absence of documented calcification in previous DFSP cases.^21^, ^24^, ^26^ While imaging findings for DFSP are nonspecific, they can aid in the assessment of complex cases and offer insight on tumour size and extent for preoperative planning.

### PET‐CT

PET‐CT combines the functional and structural insights of PET and CT scans, respectively, to assist in accurate staging, monitor treatment response, and detect recurrent disease early on.^27^ It utilizes ^18^F‐Fluorodeoxyglucose (^18^F‐FDG), a glucose analogue, to assess tissue glucose metabolism, making it a valuable imaging tracer in oncology.^27^

The use of ^18^F‐FDG PET‐CT for DFSP is limited to case reports but has shown potential in managing DFSP. For instance, Kashyap et al.^28^ reported a case where DFSP recurred a second time with fibrosarcomatous changes and extensive metastases, detected by ^18^F‐FDG PET‐CT. Subsequent treatment with Imatinib demonstrated complete metabolic resolution observed on PET scan, but only a partial response on CT, indicating significant metabolic improvement but with delayed morphological changes. Considering that over 90% of DFSP cases involve a chromosomal translocation between 17 and 22, the response seen on ^18^F‐FDG PET‐CT in this patient—who also had this translocation—implies its potential as a surrogate marker for therapeutic effectiveness in patients with similar genetic alterations.^28^

Other reports have highlighted the role of ^18^F‐FDG PET‐CT in identifying distant metastases and tumour staging, recurrence detection, and assessing tumour response to imatinib.^29^, ^30^, ^31^, ^32^, ^33^, ^34^ However, the literature does not offer conclusive guidance regarding the indications, timing, or overall role of FDG PET‐CT in the staging and surveillance of DFSP patients.^29^, ^30^, ^31^, ^32^, ^33^, ^34^ While its precise role is still to be defined, FDG PET‐CT appears beneficial in complex cases by helping to delineate disease extent, anticipate tumour recurrence, and detect distant metastasis, particularly in scenarios involving advanced inoperable or metastatic disease.^29^, ^30^, ^31^, ^32^, ^33^, ^34^, ^35^

### MRI and HR‐MRI

MRI remains the preferred imaging modality for soft tissue tumours due to its high soft tissue contrast resolution.^6^ MRI protocol includes three sequences: T1‐weighted (TW‐1), T2‐weighted (TW‐2), and short tau inversion recovery sequence (STIR). On T1‐W sequences, DFSP generally has a homogenous appearance with signal intensity similar to skeletal muscle, and lower than subcutaneous fat.^21^, ^23^, ^36^, ^37^ However, cases with signal intensity lower than skeletal muscle, and less frequently, higher than skeletal muscle have also been documented.^37^, ^38^, ^39^ On T2‐W sequences, DFSP usually shows elevated signal intensity, ranging from hyperintense to isointense relative to fat, with some lesions displaying intermediate or low intensities.^21^, ^23^, ^26^, ^37^

The STIR sequence, which suppresses fat signal, helps differentiate DFSP from surrounding fat when T2‐W sequences are insufficient.^6^, ^21^, ^23^, ^37^, ^40^

DFSP demonstrates varying degrees of contrast enhancement upon intravenous gadolinium administration, ranging from homogeneous to heterogeneous, and mild to pronounced enhancement.^16^, ^21^, ^26^, ^37^, ^41^ Hakozaki et al.^42^ suggest a pathological correlation between the degree of MRI enhancement in DFSP and its histological features, showing that pronounced MRI enhancement is associated with high‐grade sarcomatoid characteristics. Additionally, Serra‐Guillén et al.^43^ found MRI superior to clinical palpation in determining the infiltration depth of primary DFSP, achieving a sensitivity of 67% and specificity of 100%, surpassing palpation's 58% sensitivity and 90% specificity. Nonetheless, MRI's effectiveness diminishes when assessing tumours in the head, neck, and upper thorax due to challenges in accurately evaluating depth, persistence, and lateral spread.

Tumour recurrence in DFSP typically presents as a distinct nodule or mass with prolonged T1‐W and T2‐W relaxation times.^21^, ^23^, ^26^, ^40^ Specific MRI features, such as increased T2‐W intensity, enhancement on T1‐W images, and nodules within the tumour capsule suggest a higher risk of recurrence. In contrast, well‐defined borders are generally associated with lower recurrence rates post‐surgery.^26^, ^37^

HR‐MRI enhances traditional imaging by providing detailed visualization of small cutaneous lesions (1–2 mm) through multiplanar and multidirectional approaches.^44^, ^45^ Dynamic contrast‐enhanced (DCE)‐MRI, a technique assessing perfusion and vascular permeability, is widely utilized in cancers such as squamous cell, breast, and prostate cancer.^45^, ^46^, ^47^ Additionally, HR‐MRI offers a notable advantage through fat saturation (FS)‐sequence images, notably FS‐enhanced‐T1‐W imaging, enabling clear visualization of peripheral adipose tissue infiltration.^44^

Yu et al.^44^ assessed the efficacy of DCE‐HR‐MRI in diagnosing DFSP in 29 clinically suspected cases. They identified various lesions, including DFSP (*n* = 7), dermatofibroma (*n* = 9), keloid (*n* = 12), and nodular fasciitis (*n* = 1). DFSP lesions typically appeared as irregular masses, predominantly hyperintense on T2‐W imaging (5/7, 71.4%) and isointense on T1‐W imaging (6/7, 85.7%). Two cases showed mixed intensity due to bleeding and sarcomatous transformation. Enhancement was significant and homogeneous in 85.7% of cases, with deep fascia involvement in 57.1% and subcutaneous fat in 42.9%. Quantitative DCE‐MRI analysis revealed high neovascularization and endothelial damage in DFSP, evidenced by the highest mean values of Ktrans (1.01 ± 0.56) and Kep (1.58 ± 0.83). DFSP also demonstrated the highest extracellular volume fraction (0.70 ± 0.14) and initial area under the curve (44.87 ± 17.97), indicative of pronounced tissue necrosis, dense tumour cells, and rich blood supply.^48^, ^49^

Overall, an MRI is recommended for preoperative evaluation, surgical planning, and follow‐up for the recurrence of DFSP.^42^, ^50^ For pre‐surgical planning, MRI is reserved for large (>5 cm) or locally recurrent tumours. The integrative imaging capability of HR‐MRI proves advantageous, particularly for tumours infiltrating deep fascia and muscles, allowing precise depth measurement with minimal distortion of peripheral anatomy.^44^ Preoperatively, DCE‐HR‐MRI's high‐resolution depiction of DFSP infiltration into surrounding fat provides robust information and support for implementing surgical excision. In contrast, imaging methods such as the US struggle to simultaneously display such detailed features. Considering Yu et al.^44^ was the only study that reported on using this DCE‐HR‐MRI for DFSP, more studies are needed to determine its efficacy.

### MRS

MRS serves as a valuable adjunct to MRI, providing non‐invasive insights into tissue characteristics. While MRI uses hydrogen proton signals for anatomical imaging, proton MRS (1H‐MRS) determines brain metabolite concentrations.^51^ Choline, with nine magnetic‐equivalent protons, allows detectable measurements even in trace amounts.^52^ Malignant tumours, characterized by elevated cell turnover, show increased choline concentrations, detectable by 1H‐MRS.^53^, ^54^ In contrast, benign tumours, with lower cell turnover, lack a prominent choline peak on 1H‐MRS.^55^ Studies using 1.5 or 3 T MR equipment have shown 1H‐MRS can differentiate between malignant and benign soft tissue tumours.^56^, ^57^, ^58^

The role of 1H‐MRS in diagnosing DFSP is limited, but a few studies have shown promise when used adjunctively with MRI. For soft tissue tumours, 1H‐MRS has demonstrated a sensitivity of 94.7% and a negative predictive value of 93.8%.^58^ Notably, despite being a malignant tumour, studies by Djilas‐Ivanovic et al. and Russo et al.^10^, ^58^ have shown that DFSP does not exhibit a choline peak, unlike other soft tissue tumours. Djilas‐Ivanovic et al.^10^ suggest that MRI features characteristic of DFSP, combined with the absence of a positive choline peak on 1H‐MRS, could help in diagnosing DFSP. However, it is important to note that histology remains the gold standard for diagnosing DFSP, with MRS serving only as a possible adjunct tool.

### OCT

High‐definition‐OCT (HD‐OCT) is a non‐invasive imaging technique that has gained recent popularity. Unlike traditional histopathology, which requires the removal of a tissue specimen for microscopic examination, HD‐OCT can provide real‐time, in‐situ images of the tissue.^59^ Its application in the context of DFSP is not extensively documented in the literature, but one case did report using the technology following a biopsy‐confirmed diagnosis of DFSP.^60^ The tumour was assessed using slice mode, revealing dermal thickening and dermal–epidermal junction (DEJ) loss. In en‐face mode, disorganized dermal layering, replacement of collagen bundles by less‐reflective elongated cells, and widened vessels were noted. Contrastingly, the skin next to the tumour displayed normal tissue architecture. This case proposed that distinct morphological characteristics of DFSP that may not be seen in conventional histopathology may be identified using HD‐OCT, indicating its use as an adjunct to the traditional diagnostic methods.^60^

Although data on HD‐OCT application in DFSP cases is limited, this case highlights its ability to accurately distinguish DFSP from normal skin histological features. This emphasizes its potential as a reliable adjunctive tool for preoperative assessment confirming precise surgical margins, potentially reducing relapse rates and enhancing conservative tissue‐sparing excisions.^60^

Line‐field confocal OCT (LC‐OCT), introduced in 2018, has gained increasing popularity in dermatology.^61^ By integrating the strengths of OCT and reflectance confocal microscopy (RCM), LC‐OCT provides enhanced image resolution that closely matches the clarity of RCM while surpassing conventional OCT. Additionally, it allows for the real‐time acquisition of both vertical and horizontal sections, as well as three‐dimensional imaging.^62^ There may be a role for LC‐OCT in managing DFSP, as demonstrated by Bouissingault et al.,^63^ though further studies are necessary to substantiate these findings.

### Dermatoscopy

Dermatoscopy, regarded as the dermatologist's stethoscope, is widely available, fast, cost‐effective, and relatively easy to perform.^64^ Despite its general utility, dermatoscopic findings specific to DFSP are limited in the literature.^65^, ^66^, ^67^, ^68^, ^69^, ^70^, ^71^, ^72^, ^73^, ^74^, ^75^ Some common dermatoscopic findings are the presence of vessels (81%), a pigmented network (78%), a pinkish background (66%), shiny white streaks (53%), structureless hypo‐ or depigmented areas (50%), and structureless light brown areas (44%).^74^ Additionally, DFSP most commonly displays coexistence of reticular pigmentation and unfocused and/or focused arborizing vessels. Unlike the conventional dermatoscopic rule linking a pigment network to melanocytic lesions, here the observed pigment network showed heightened pigment in the epidermal basal cells, deviating from the standard diagnostic guideline.^76^

Atypical dermatoscopic presentations of DFSP have also been documented, making dermatoscopy for DFSP even less reliable. Güngör et al.^68^ described an atrophic variant, characterized by arborizing telangiectasias against a yellowish background indicative of dermal atrophy. Ehara et al.^69^ reported a pigmented variant (Bednar tumour), which resembled a blue nevus with homogeneous black‐bluish pigmentation and white‐veil structures. Additionally, Hartmann et al.^70^ observed features similar to basal cell carcinoma, including arborizing telangiectasias and blue‐grey ovoid nests.

The role of dermatoscopy in DFSP remains unclear and under‐documented. Current evidence, primarily based on a limited number of case reports, suggests that dermatoscopy is not useful for DFSP diagnosis or management. Further research is needed to conclusively assess its accuracy and determine its diagnostic value for DFSP.

## DISCUSSION

Diagnosing DFSP can be challenging due to its rarity and varied clinical presentation. Given the characteristic histologic appearance of most DFSPs on biopsy, imaging techniques are primarily used for preoperative evaluation and surgical planning. However, US findings, when combined with patient history and physical examination, can raise clinical suspicion of DFSP, advocating for a timely biopsy to confirm diagnosis.^77^, ^78^

**FIGURE 1:**
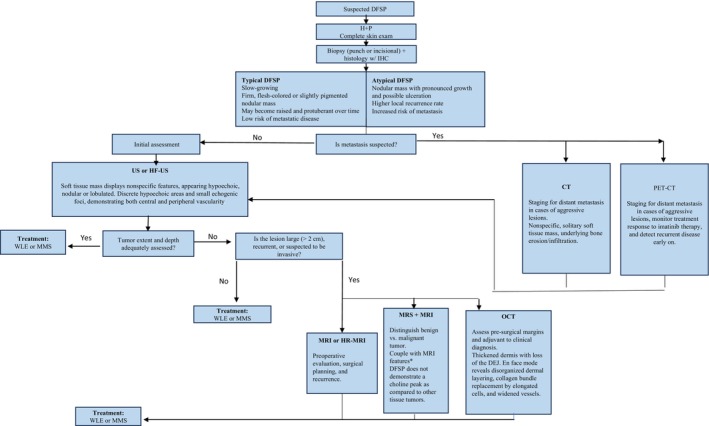
Diagnostic and treatment pathway for DFSP. CT, computed tomography; DFSP, dermatofibrosarcoma protuberans; H + P, history and physical; HF‐US, high frequency ultrasound; MMS, Mohs micrographic surgery; MRI, magnetic resonance imaging; PET‐CT, positron emission tomography‐computed tomography; US, ultrasound; WLE, wide local excision.

Currently, no standard staging system for DFSP exists; however, the primary tumour is generally considered stage I, lymph node metastasis stage II, and distant metastasis stage III.^79^ Simple clinical palpation alone is insufficient to assess tumour extension adequately. Thus, imaging tests and lymph node evaluations are used to stage the disease and plan surgical intervention. Assessing the anatomical extent of DFSP through imaging is particularly valuable in settings where MMS is not available, necessitating the use of WLE instead.^80^ Figure 1 summarizes the imaging pathway for management of DFSP.

Given their speed, accessibility, and cost‐effectiveness, US or HFUS could be prioritized for initial imaging in the management of DFSP. US can be used for pre‐operative planning to evaluate tumour size and extent, and provide biopsy guidance if needed.^1^, ^12^, ^15^ Notably, the US demonstrated superior sensitivity (81.8%) compared to MRI (67%) in determining the extent and invasion of DFSP.^15^, ^43^ The superior sensitivity of the US suggests its potential as an effective point‐of‐care (PoC) tool in the management of DFSP. Its use could streamline clinical decision‐making, reduce the reliance on multiple imaging tests, and reduce unnecessary transportation and relocation burden.^81^

Where MRI excels is in post‐operative follow‐up for detecting recurrence.^82^ Most local recurrences manifest within the first 3 years; however, between 25% and 30% of cases occur after 5 years.^83^, ^84^, ^85^, ^86^, ^87^ Consequently, lifelong follow‐up is recommended after treatment to monitor for any late‐developing recurrences. Because MRI can effectively distinguish recurrent DFSP (nodular and homogeneous configuration with well‐defined borders and marked contrast enhancement) from other findings and the surrounding tissue, a close MRI follow‐up (every 6–12 months) for the first three postoperative years can be considered, especially for aggressive or recurrent cases. Afterward, the follow‐up interval can be extended to once a year for at least 2 more years.^82^ However, MRI is not useful for detecting tumour persistence in cases of incompletely extirpated tumours.^43^

The fat infiltration detectable through HR‐MRI can help in differentiating DFSPs from cellular dermatofibromas (CDFs), as the tumour cells of CDFs may extend into subcutaneous fat, but do not infiltrate the fat septum and lobules.^88^ The distinct pattern of adipose infiltration seen in DFSP—often resembling a histopathological ‘honeycomb’ pattern—suggests invasive growth and correlates with the tumour's potential for recurrence after simple excision. This level of detail can be important for planning WLEs in complex cases where precise delineation of tumour boundaries is critical for reducing recurrence. Additionally, HR‐MRI's ability to quantitatively assess tumour microcirculation provides unique data on tumour biology, which can inform more personalized treatment strategies.^44^ When imaging features are not adequate to reach a robust conclusion, quantitative parameters could be referred. While HR‐MRI should not be considered a standard requirement for managing DFSP cases, its use can be valuable in challenging cases where traditional imaging techniques fall short. Further research is needed to define the specific scenarios in which HR‐MRI adds the most value, thereby optimizing its use in clinical practice for DFSP management.

CT is primarily reserved for advanced‐stage tumours to evaluate for underlying bone involvement and distant metastatic disease.^1^ Due to the rarity of lymphatic and hematogenous spread, a comprehensive staging workup is generally not required. It should only be considered if the patient's history and physical examination indicate potential metastasis, if there are negative prognostic histologic traits present (Ex. fibrosarcomatous changes), or in cases of tumour recurrence. Furthermore, in cases of advanced or recurrent disease, a chest CT is recommended to screen for pulmonary metastases.^79^, ^89^ While PET‐CT has been useful in identifying primary and metastatic lesions and staging disease status, its unique utility lies in the capability of ^18^F‐FDG PET/CT to assess treatment response to imatinib.^29^, ^30^, ^31^, ^32^, ^33^, ^34^ Accumulating evidence indicates that imatinib may be effective in treating advanced DFSP, with some patients achieving prolonged complete remission.^31^, ^90^, ^91^, ^92^, ^93^, ^94^, ^95^ Thus, in cases of large unresectable, recurrent, and/or metastatic DFSP where imatinib therapy may be considered, ^18^F‐FDG PET/CT has potential in guiding treatment adjustments and enhancing treatment outcomes. Similar to its role in nonsmall cell lung cancer, an initial response evaluation PET could potentially predict outcomes in DFSP patients.^28^

While imaging modalities can be useful for managing DFSP, they come with limitations.

US effectiveness heavily depends on the operator's skill, which, due to DFSP's rarity, may lead to misdiagnosis if sonographers are unfamiliar with its typical presentations. Thus, providers must recognize their limitations and engage in continuous professional development to maintain accuracy and competency in using the US.^81^ US images also require careful correlation with clinical and histological findings due to their often‐nonspecific nature. CT, especially when integrated with PET‐CT, presents risks associated with radiation exposure. This is particularly concerning for young patients or those requiring multiple follow‐up scans, as the cumulative radiation dose can contribute to the risk of developing secondary malignancies.^96^ Moreover, CT struggles to differentiate between certain soft tissue characteristics without contrast enhancement. MRI, despite offering detailed soft tissue visualization, is more costly and less accessible than other modalities. HD‐OCT and LC‐OCT, while providing high‐resolution images, also has its limitations, such as restricted depth penetration, limiting visualization of deeper skin structures. Motion artefacts can degrade image quality, particularly in areas prone to movement, and interpreting complex OCT images can be challenging and dependent on the provider's experience. Additionally, the cost and availability of OCT systems restrict widespread clinical use, and the lack of standardized protocols for OCT imaging in dermatology affects consistency and reliability.^60^

Clinical observation alone is inadequate for preoperative assessment of tumour extension in DFSP. Non‐invasive imaging presents as a valuable tool for managing individuals with DFSP, especially in atypical, aggressive, and recurrent cases. Although various imaging modalities have been documented as useful, there is still limited evidence on which are most effective for DFSP. Current literature often describes the imaging characteristics of DFSP but seldom compares the efficacy of different modalities. Further research is needed to evaluate and compare the accuracy of these imaging techniques in the context of DFSP.

## CONCLUSION

DFSP is a rare soft tissue tumour characterized by asymmetrical and poorly defined growth. Diagnosis is challenging due to its resemblance to other benign skin conditions, and management is further complicated by its infiltrative growth pattern and high risk of local recurrence. Thorough preoperative planning is important, particularly in healthcare settings where MMS is unavailable. A comprehensive radiological evaluation, including US, CT, and MRI can be useful in assessing the anatomical extent of DFSP, with the US emerging as a sensitive and cost‐effective initial imaging modality. HR‐MRI offers promise for accurate preoperative assessment, especially with its ability to depict adipose tissue infiltration and measure exact depth values. Additionally, the role of emerging technologies such as MRS and OCT is being explored, showing potential for enhancing DFSP management. However, given the limitations and varying efficacies of each imaging technique, a combination of modalities, informed by clinical and histological correlations, may provide the most comprehensive evaluation. Further comparative studies are needed to refine imaging strategies and enhance the management of DFSP.

## CONFLICT OF INTEREST STATEMENT

The authors YAA, AP, JT, and NCZ declare that they have no affiliations with or involvement in any organization or entity with any financial interest in the subject matter or materials discussed in this manuscript.

## Data Availability

Data sharing is not applicable to this article as no new data were created or analyzed in this study.
